# The SARS-CoV-2 crisis and its impact on neglected tropical diseases: Threat or opportunity?

**DOI:** 10.1371/journal.pntd.0008680

**Published:** 2020-09-21

**Authors:** Claire Chaumont, Kimberly Kamara, Elisa Baring, Karen Palacio, Ana Power, Warren Lancaster

**Affiliations:** 1 The END Fund, New York, New York, United States of America; 2 Harvard T. H. Chan School of Public Health, Boston, Massachusetts, United States of America; 3 Graduate School of Public Health and Health Policy, City University of New York, New York, New York, United States of America; KU Leuven, BELGIUM

## Introduction

The current global priority is to protect people from attaining the COVID-19 infection and to attend to those infected, resulting in the disruption of other activities of the health sector, such as neglected tropical disease (NTD) control and elimination programs, which, across the globe, have postponed mass drug administration (MDA) campaigns. The impact of this disruption, combined with that of the pandemic, will ripple for years to come. This commentary reflects on how the current crisis modifies the future of the NTD sector focused on the five diseases treated through preventative chemotherapy (often called PC-NTDs): soil-transmitted helminths, schistosomiasis, lymphatic filariasis, onchocerciasis, and trachoma.

## Resurgence of infections

The most obvious impact of the crisis on NTD programs relates to its effect on infection prevalence due to delayed mass drug administration campaigns. The World Health Organization (WHO) developed a new roadmap that was meant to officially launch in June 2020 that includes specific disease targets to control and eliminate NTDs by 2030 [1]. With SARS-CoV-2, not only is the launch of the roadmap postponed, but all NTD activities were postponed to prevent risk of additional transmission of COVID-19 [2]. With the moratorium slowly being lifted, tools and guidelines are emerging to help countries undertake the necessary steps for NTD interventions to not fall too far behind its disease control and elimination goals.

All five PC-NTDs require annual mass treatment with high treatment coverage to reduce infection rates. Delays or cancelation of such campaigns can lead to infection resurgence. A recent modelling exercise has shown that these delays will particularly impact diseases with short elimination timelines (three to five years), such as trachoma or lymphatic filariasis, and areas with high levels of schistosomiasis infections where interruption of set cycles could erase years of effort [3]. Delays and interruptions to treatment cycles are not unknown to the NTD space, due to setbacks in the supply chain, unseasonal rains, conflicts, political campaigning, or even reduced funding. NTD programs are remarkably adept at making the necessary accommodations to adjust for such delays [4]. Regardless, many countries may need to “catch up” with intermediate rounds of MDAs to mitigate the effect of the enforced pause on infection rates in communities. In addition, delays to monitoring and evaluation activities may further impact the ability of the community to allocate stretched resources as efficiently as possible. This is especially relevant for schistosomiasis, considering recent global efforts to develop more precise maps to identify hot spots.

## Political and funding landscape for NTDs

Prior to the SARS-CoV-2 outbreak, NTD prevention through MDA was described as the “best buy in public health” [5]. Despite the high Disease-Adjusted Life Years (DALYs) burden, the five PCT diseases risk being deprioritized if resources are stretched due to their low mortality rate when compared to diseases such as malaria [6]. The prioritization of SARS-CoV-2 stalled NTD programming and stretched staff, including community health workers (CHWs), who are often redeployed to support other pressing health matters. Some NTD funding, both internal and external, was repurposed in the short term in order to contribute to the SARS-CoV-2 response for acute services or products, and it is unclear whether such funds will be replenished [7]. Long-term funding for NTDs may also be impacted. This is especially true if a resurgence of infection pushes elimination goals further in the future, if the costs of NTD interventions significantly increase due to the inclusion of needed safety measures, or if additional MDA rounds are needed to limit disease resurgence.

## NTD service delivery

The SARS-CoV-2 crisis will also profoundly change the design and delivery of NTD programs. Shifts in human capital availability and transmission dynamics of SARS-CoV-2 will require national NTD programs to adopt different implementation approaches to ensure availability and use of appropriate personnel, as well as preventive measures, including social distancing, safe hygiene practices, and face masks and/or coverings.

Health personnel at national and subnational levels are joining the pandemic response. Human resources for NTD activities are thus shifting due to economic crisis and health system priorities. This will result in reduced capacity and turnover to focus on restarting the planning of interventions, both for preventive and curative services. In addition, preventative chemotherapy has historically relied on community drug distributors, voluntarily or paid. In the future, there may be less willingness for individuals in these roles to support MDA due to an overwhelmed system and economic strains that stymie volunteerism. A reevaluation of human capacity needs will be required to determine the appropriate approach to restart activities.

The increased need for social distancing will also modify NTD program delivery. House-to-house treatment may need to be favored over fixed-point distribution, to minimize people coming together in crowds. This will impact how community drug distributors or teachers are mobilized for distribution (e.g., teachers usually involved in school-based distribution may have to reach children in their houses). When distribution must be organized at fixed points, rules limiting the number of people gathering will have to be put in place. In addition, new protocols may need to be put into place for morbidity management or surgical activities to ensure patients can safely receive these services.

## Community acceptance and social mobilization

Reengaging communities on access to free treatment and case management services will be another hurdle for NTD programs to overcome. MDA campaigns are dependent on a high level of community acceptance to ensure high coverage levels of the campaign. However, amidst injunctions of social distancing, communities may be skeptical of gathering. An overall distrust of health services and personnel may exist, a situation that arose post the Ebola outbreak in West Africa. In 2015, the Ministry of Health and Social Welfare (MoHSW) of Liberia conducted a Post-Ebola NTD Readiness Assessment, which outlined the need to improve community awareness and understanding of the treatment [8]. These lessons guided the scale-up of NTDs post-Ebola and the revisions to social mobilization to ensure the success of the program. As NTD programs assess restarting activities, revising communication strategies will be key for community and health worker uptake.

## What comes next?

The SARS-CoV-2 pandemic seriously threatens the gains made by NTD programs in recent years. At the same time, the sector is endeavoring to use this moment to think more synergistically to adapt this extraordinary vertical campaign platform to new circumstances to further serve populations affected by NTDs and fight the pandemic along the way.

First, the way the NTD sector responds to delays is an opportunity to modify or accelerate treatment strategies (for example, through additional MDA rounds, expanded treatments, or more targeted services for high-transmission areas). This goes along with the growing realization that a greater investment should be made in securing consistently high coverage and intake of preventative chemotherapy. Leveraging and learning from other delivery platforms’ experiences during other campaigns, such as polio, malaria, or Human African Trypanosomiasis (which have all leveraged geospatial data to identify untreated areas, map out all settlements and plot efficient routes, and used GPS to confirm delivery), could also strengthen the NTD platform. The need to “catch up” because of COVID-19–related delays may spur further treatment innovations or adaptations, including in research & development for drugs and diagnostics.

In parallel, there is an opportunity to try different delivery models to ensure the success of NTD activities restarting while mitigating potential risks. Control and elimination activities for the five PCT NTDs traditionally utilize a campaign model through community- and school-based platforms. An alternative could be returning to a former approach used for the control of onchocerciasis, Community Directed Treatment with Ivermectin (CDTI), in which the treatment was conducted by community members over a longer period of time [9]. This approach could better mitigate the risk of SARS-CoV-2 transmission, but community acceptance and costs would need to be carefully considered, as well as its potential impact on coverage. Another approach would be to explore further the integration of NTDs into routine primary health care services, such as the delivery of drugs in antenatal care clinics or nutrition programs. Coordinating treatment alongside food distribution efforts, particularly for schistosomiasis control, could also be a positive outcome in a time when economic crisis may lead to further food insecurity for vulnerable populations at risk of NTDs. Taking the medication with food would ease any side effects of the drug Praziquantel and could increase overall compliance. There are also opportunities for expanding the use of Mhealth technology for payment transfer, monitoring, and supervision of NTD activities to maximize “touchless” approaches.

Finally, restarting NTD programming is an opportunity to reconsider social mobilization strategies. Information campaigns will require additional efforts put towards discouraging mass gathering for treatment and safety measures for health or community worker-led treatments. It will be critical to educate drug distributors and the communities on COVID-19 symptoms to ensure they are not mistaken for serious adverse events. Sensitization of treatment will also require additional channels of communication as the predominant health communication is currently focused on pandemic-related news. Programs should consider conducting surveys to better understand if there is a need to increase efforts to mobilize and generate demand among communities.

## Conclusion

The COVID-19 pandemic will have a long-lasting economic, social, and health impact across the globe. In the field of NTDs, it may lead to reinfections due to delayed care. In this brave new world, the NTD community has a responsibility to advocate for continued prioritization of NTDs on the global health agenda, in alignment with the likely transformed political and funding landscape. This could be a pivotal moment to further strengthen health systems with embedded horizontal platforms for NTD prevention.
